# Postpartum Management of Placenta Accreta with Transcervical Radiofrequency Ablation for Fertility Conservation

**DOI:** 10.3390/jcm15083066

**Published:** 2026-04-17

**Authors:** Nicole Santella, David Toub, Leslie Hansen Lindner

**Affiliations:** 1 Wake Forest University School of Medicine, Winston-Salem, NC 27101, USA; 2 Independent Gynecology and Medical Affairs Consultant/Researcher, Wyncote, PA 19095, USA; dtoub@mac.com; 3 Atrium Health, Carolinas Medical Center, Charlotte, NC 28204, USA; leslie.hansen-lindner@advocatehealth.org

**Keywords:** placenta accreta, radiofrequency ablation, fertility, hysteroscopy, postpartum

## Abstract

**Background**: Placenta accreta spectrum (PAS) refers to the abnormal placental implantation into the uterine wall, and its incidence is rising in parallel with increasing cesarean deliveries and myomectomies. PAS carries high maternal risks, including hemorrhage, shock, and death. Management involves either a hysterectomy or conservative approaches to preserve fertility that come with higher risks of maternal morbidity and mortality. Radiofrequency ablation (RFA) is a well-established modality for treating soft tissue tumors, but its use for PAS is not well studied. **Case**: We report a case of successful postpartum treatment of placenta accreta with transcervical radiofrequency ablation, which preserved uterine integrity and resolved significant bleeding without postoperative complications. **Conclusions**: Transcervical RFA may offer a safe and minimally invasive treatment for placenta accreta that conserves the uterus and may maintain reproductive capacity.

## 1. Introduction

Placenta accreta spectrum (PAS) is defined by three grades of abnormally adherent placenta or invasive placenta: grade 1 is when placenta is attached directly to the surface of the middle layer of the uterine wall without invasion; grade 2 includes placental invasion into the myometrium; and grade 3 involves placental invasion that may reach the surrounding pelvic tissues, vessels, and organs [1]. This nomenclature replaces the older terminology for varying degrees of abnormal placental adherence, namely placenta accreta, placenta increta, and placenta percreta (in order of invasion). The main differential diagnostic consideration is retained placenta, in which the placenta is not spontaneously expelled within 30 min of delivery and is readily removed either manually or with curettage. Placenta accreta can be further characterized as total, partial, or focal depending on the amount of placental tissue adhered to the myometrium [1]. The pathogenesis of PAS is not fully understood, but it is believed to relate to trauma or abnormality of the endometrium and myometrial interface, leading to the failure of normal decidualization in the area of scarring, which allows placental villi and trophoblast infiltration [2]. The incidence of placenta accreta has increased significantly since the 1970s, ranging from 1.7 to 4.6 per 10,000 deliveries [2,3]. This increased incidence is believed to be related to the increased number of cesarean sections, which are a known risk factor for placenta accreta. PAS results in increased morbidity and mortality in pregnant patients due to increased risks for hemorrhage, coagulopathy, shock, urinary tract injury, and death [2,4]. Current management of placenta accreta includes gravid or interval hysterectomy versus conservative management, such as leaving the placenta in situ or performing wedge resection, though this can result in delayed infections or bleeding [3]. There are no comparative studies between or among uterine artery embolization (UAE), operative hysteroscopy, and expectant management for placenta accreta, with the literature for resection and/or UAE comprising case reports and small case series, none of which are definitive. To date, only radiofrequency ablation has emerged as more than a niche thermal ablative treatment for placenta accreta, as neither microwave ablation nor cryoablation have been studied in this context.

Radiofrequency ablation (RFA) has been used for either the definitive treatment or palliation of benign and malignant soft tissue tumors, including hepatocellular carcinoma, uterine fibroids, and adenomyosis [5,6,7,8,9,10,11,12]. RFA uses electrodes directly inserted into a target lesion, generally under ultrasound or laparoscopic guidance, to generate heat and induce thermal fixation and coagulative necrosis in the target lesion [12]. There has been very limited data regarding the use of RFA to treat placenta accreta, and this has mainly comprised animal, in vitro, and solitary clinical case reports [13,14].

The Sonata^®^ system (Hologic, Inc.; Marlborough, MA, USA) is an FDA-cleared and CE-marked (Conformité Européene) device that is indicated for diagnostic intrauterine imaging and transcervical treatment of symptomatic uterine fibroids. The Sonata system is currently the only commercially available device in the United States for transcervical fibroid ablation (TFA). The Sonata system includes integrated intrauterine ultrasound along with a radiofrequency electrode array to perform volumetric RF ablation of fibroids. The fibroid types treatable with Sonata include FIGO types 1, 2, 3, 4, 5, and 2–5 (transmural) fibroids, most of which are not amenable to hysteroscopic resection [8]. Because Sonata includes a graphical guidance system (SMART^TM^ Guide) that displays where the ablation is taking place and includes a Thermal Safety Border beyond which there is no material risk of thermal effects, trained gynecologists can safely ablate appropriate fibroids throughout the uterine corpus. The use of RFA in this procedure does not involve the peritoneal cavity or uterine serosa and therefore allows for a minimally invasive, transcervical method of ablating uterine fibroids [8]. The use of this device for PAS remains outside of the approved device labeling. The Sonata system has been described in additional detail elsewhere [8,10,15,16,17].

We present the case of a 43-year-old woman who underwent transcervical radiofrequency ablation of placenta accreta using the Sonata system in the postpartum setting, resulting in the cessation of bleeding and uterine conservation without postoperative complications.

## 2. Case

A 43-year-old gravida 2, para 1 patient with monochorionic diamniotic twins conceived spontaneously and received routine prenatal care. Her obstetrical history consisted of a prior vaginal delivery with no prior hysterotomies, nor a history of dilation and curettage. She was delivered via a primary low transverse cesarean section at 36 weeks due to polyhydramnios and breech presentation. The placenta was carefully inspected at the time of delivery and noted to be intact. The delivery was complicated by postpartum hemorrhage responsive to multiple pharmacologic agents, with an estimated blood loss of 1250 mL. She was discharged on postpartum day 3.

The patient presented to the Emergency Department (ED) 35 days postpartum for heavy bleeding. She was tachycardic and hypotensive, with significant abdominal tenderness and spontaneous passage of blood clots from her vagina. The lab results revealed a hemoglobin of 10.7 g/dL, hematocrit 33, and a platelet count of 241. She received uterotonics, antifibrinolytics, and one unit of packed red blood cells (PRBCs) while in the ED. A bedside ultrasound showed an enlarged uterus with probable retained products of conception (RPOC). She was taken to the operating room (OR) for a suction curettage to remove the RPOC. Methergine was utilized and hemostasis was assured. She was discharged and sent home on postoperative day 1. The histopathology results returned as multiple portions of blood clot, decidua, and villous tissue.

**Figure 1:**
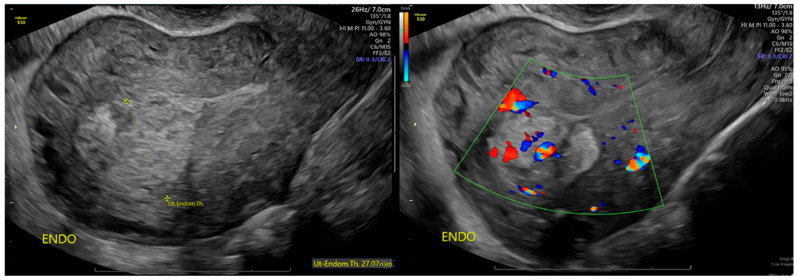
Ultrasound obtained after initial vacuum curettage showing heterogenous endometrium measuring 27 mm with Doppler ultrasound consistent with retained products of conception.

The patient presented to the office 22 days after her curettage for a postoperative visit and noted the continued presence of light vaginal bleeding. An ultrasound showed a heterogenous endometrium measuring 27 mm, with a color flow Doppler study suggesting persistent RPOC (Figure 1). Labs were drawn at this time, and the patient was noted to be anemic with a hemoglobin of 8.8 g/dL, hematocrit 29, and a platelet count of 320. She was taken to the OR 3 days later for a hysteroscopy, dilatation and curettage (D&C), and the removal of RPOC.

At the time of surgery, the placental tissue was visualized in the anterior uterine wall. RPOC removal was performed with a transcervical tissue removal system, resulting in incomplete excision of RPOC due to bleeding and impaired visualization. Curettage under ultrasound guidance only returned a small amount of additional tissue. Given persistent RPOC despite multiple interventions, the diagnosis of focal placenta accreta was suspected.

**Figure 2:**
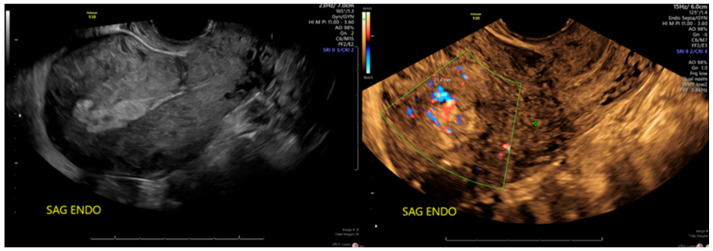
Ultrasound obtained 2 weeks after hysteroscopy and dilatation and curettage, showing persistent 2.5 × 2.0 cm area of thickened tissue with irregular borders and areas of vascularity suggesting focal placenta accreta.

A repeat ultrasound 2 weeks postoperatively showed a persistent 2.5 × 2.0 cm area of thickened tissue with irregular borders and prominent areas of vascularity, consistent with focal placenta accreta (Figure 2). The patient expressed a strong desire to avoid a hysterectomy and inquired about the possibility of a uterus-conserving surgical procedure. The use of the Sonata device to treat placenta accreta is not within the approved labeling, but it was regarded to be a very reasonable approach to potentially minimize bleeding and improve visualization at the time of hysteroscopic resection. The ability to sonographically outline the abnormal tissue and safely delineate the myometrium and serosa as a means of treating any unresectable accreta was proposed, as an operative hysteroscopy alone was not deemed safe or practical for treating deeper placental tissue fragments.

**Figure 3:**
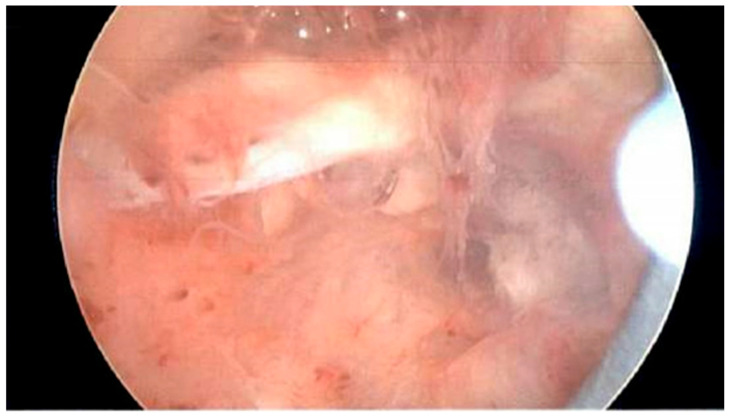
Hysteroscopy at the time of radiofrequency ablation procedure, showing a 2.0 × 2.5 × 2.5 cm area of irregular myometrium with no discernable plane corresponding to the ultrasound visualized hyperechoic region on the left anterior fundus that was targeted with Sonata device for ablation.

The patient was taken to the operating room under general anesthesia. Using intrauterine sonography, a 2.0 × 2.5 × 2.5 cm hyperechoic region was noted on the left anterior fundus and confirmed with the hysteroscopy (Figure 3). Transcervical RFA was performed, with three overlapping ablations performed over 4.5 min. The Sonata system utilizes a generator that modulates power delivery to maintain the temperature at the needle electrode tips at a constant 105 degrees Celsius and can deliver up to 150 Watts, with a mean wattage around 30–40 Watts. The placental tissue was ablated to its edge. A resectoscope was then inserted, and the placental tissue was progressively excised from the surrounding myometrium. Repeat intrauterine sonography was performed to confirm the lack of persistent placental tissue within the myometrium. The total procedural duration was 50 min, and the estimated blood loss was 20 cc. No perioperative complications were observed or reported.

**Figure 4:**
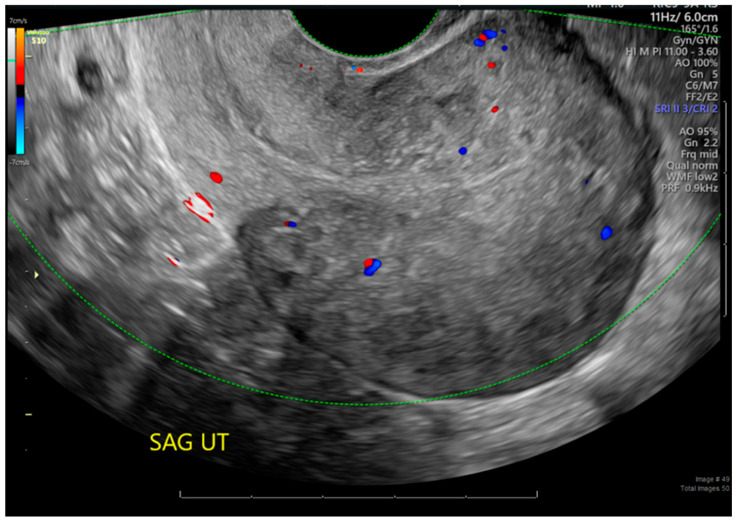
Two-week follow-up ultrasound showing endometrial width of 7.1 mm without abnormal Doppler flow, indicating resolution of accreta with Sonata-guided ablation.

The patient’s abnormal bleeding was resolved within 2 weeks. Pathology revealed an implantation site and placenta accreta in the resected myometrium. Follow-up sonography showed a normal myometrial thickness and an endometrial width of 7.1 mm without abnormal Doppler flow (Figure 4). The patient has had no further episodes of bleeding in the 10 months since the procedure.

## 3. Discussion

Due to higher prevalences of cesarean sections and myomectomies, including multiple repeat hysterotomies, the incidence of placenta accreta spectrum has been increasing, with primary management directed at a hysterectomy, either at delivery or at an interval post-delivery [18,19,20]. Conservative management options include leaving the placenta in situ; manual removal of the placenta; transcervical resection of placental remnants along with the invaded myometrium; and uterine devascularization through UAE [21]. The PACCRETA study compared severe maternal outcomes for women with PAS treated with hysterectomies versus conservative management (placenta left in situ). The study demonstrated lower rates of total blood loss, adjacent organ injury, transfusion, and non-hemorrhage-related severe maternal morbidity in the conservative management group, but high levels of endometritis, the need for UAE, and readmission within 6 months [3]. Conservative pharmacologic measures are also limited due to their risks of failure, infection, and incomplete placental involution. This suggests a need for better techniques for conservative management, such as transcervical RFA, as utilized in our patient.

In the case of our patient, it was made clear to her that the use of RFA for treatment of placenta accreta was outside of the indication for transcervical RF ablation with the Sonata system, and the benefits were not known. As standard therapies had failed her, she made an informed decision to proceed. The safety of the procedure was ensured by the SMART^TM^ Guide, in which the ablation region, as well as a thermal safety margin (both of which vary depending on the graphically selected ablation diameter), showed the gynecologist where the actual ablation will take place (the Ablation Zone) and, importantly, a region beyond which there was no significant thermal heating (the Thermal Safety Border).

There have been limited reports of radiofrequency energy used for the management of placenta accreta. Katsogiannou and colleagues described a case with incomplete placental delivery during a vacuum-assisted vaginal delivery [14]. The placenta was left partially in situ, which resulted in heavy postpartum bleeding managed via UAE. The patient presented 7 days after delivery with a new hemorrhage, and percutaneous RF ablation of hypervascularized areas of retained placenta accreta was performed under anesthesia. Unfortunately, the patient had a recurrent hemorrhage 13 days later and underwent repeat UAE and a subsequent hysterectomy.

Another emerging minimally invasive treatment for placenta accreta is hysteroscopic resection after high-intensity focused ultrasound (HIFU), which uses ultrasound waves to penetrate the abdominal wall and focus on the placental tissue [22,23]. The acoustic waves are converted to thermal energy, which causes the target tissue to undergo coagulative necrosis, and ultrasound imaging is used throughout the procedure to monitor the tissue response [23]. A study was conducted to compare early and late hysteroscopic resection of placenta accreta after HIFU, noting that 55% of the early group and 26.1% of the late group required two sessions of hysteroscopies [24]. One patient was noted to suffer from an intrauterine infection and another from major bleeding, highlighting the limitations of conservative management of placenta accreta. The study was also limited in its ability to evaluate long-term pregnancy outcomes because of a small number of patients desiring a future pregnancy and the lack of long-term follow up. Though HIFU remains a possible treatment for placenta accreta, it is limited by the need for multiple procedures and further investigation into the optimal timing between HIFU and hysteroscopic resection. Radiofrequency ablation may offer the advantage of achieving definitive treatment in a single session.

Our case is the first to describe the successful treatment of placenta accreta using transcervical, intrauterine ultrasound-guided RFA, followed by concomitant hysteroscopic resection, as well as the first successful management of ongoing bleeding from placenta accreta using RF ablation in the postpartum state, occurring 83 days after the time of delivery and after the patient failed treatment with both suction curettage, hysteroscopic resection, and sharp curettage.

This successful treatment of placenta accreta highlights a potential, novel approach for conservative PAS management. In appropriate cases, targeted RFA may offer a means for conserving the uterus while avoiding the limitations of current conservative measures, such as failure, incomplete placental involution, and infection [25]. Transcervical RFA, combined with resectoscopy, was successful in the postpartum period for our patient with a localized area of placenta accreta. Nonetheless, these results from a single case must be interpreted with caution, given the lack of clinical trials, controlled or otherwise, regarding the use of RFA to manage placenta accreta spectrum. Transcervical RFA allowed for the successful ablation and resection of highly vascular tissue with minimal blood loss and complete symptom relief. While we remain optimistic, future fertility after RFA and conservative management of placenta accreta will require additional study to determine its safety and optimal mode of delivery, as is true for the indicated use of TFA for women with symptomatic fibroids. In a TFA case series of 89 pregnancies among 72 women with uterine fibroids treated with the Sonata system, there were no instances of uterine rupture, PAS, or stillbirth [26]. However, the authors noted that this represented a limited dataset and could not establish the safety or optimal delivery route for women who had undergone RFA for symptomatic uterine fibroids.

## 4. Conclusions

Transcervical RFA may offer a minimally invasive, low-risk treatment of placenta accreta that conserves the uterus and allows for the consideration of future fertility. Larger case series and comparative trials are necessary to establish the optimal timing of accreta treatment with RFA, treatment parameters, and how RFA compares with other uterine-conserving and radical (e.g., gravid hysterectomy) options.

## Data Availability

The clinical data presented in this article are not readily available to maintain privacy of the treating physicians and their patient.

## Abbreviations

The following abbreviations are used in this manuscript:

PAS	Placenta Accreta Spectrum
RFA	Radiofrequency Ablation
UAE	Uterine Artery Embolization
TFA	Transcervical Fibroid Ablation
ED	Emergency Department
PRBCs	Packed Red Blood Cells
RPOC	Retained Products of Conception
OR	Operating Room
D&C	Dilation and Curettage
